# Nicotine, Auditory Sensory Memory, and *sustained* Attention in a Human Ketamine Model of Schizophrenia: Moderating Influence of a Hallucinatory Trait

**DOI:** 10.3389/fphar.2012.00172

**Published:** 2012-09-28

**Authors:** Verner Knott, Dhrasti Shah, Anne Millar, Judy McIntosh, Derek Fisher, Crystal Blais, Vadim Ilivitsky

**Affiliations:** ^1^Institute of Mental Health Research, University of OttawaOttawa, ON, Canada; ^2^Neuroscience Program, Department of Cellular and Molecular Medicine, University of OttawaOttawa, ON, Canada; ^3^School of Psychology, University of OttawaOttawa, ON, Canada; ^4^Institute of Cognitive Science, Carleton UniversityOttawa, ON, Canada; ^5^Royal Ottawa Mental Health CentreOttawa, ON, Canada; ^6^Department of Psychology, Mount Saint Vincent UniversityHalifax, NS, Canada

**Keywords:** nicotine, nicotinic acetylcholine receptor, *N*-methyl-d-aspartate receptor, sensory memory, mismatch negativity, attention, rapid visual information processing task, schizophrenia

## Abstract

**Background:** The procognitive actions of the nicotinic acetylcholine receptor (nAChR) agonist nicotine are believed, in part, to motivate the excessive cigarette smoking in schizophrenia, a disorder associated with deficits in multiple cognitive domains, including low-level auditory sensory processes and higher-order attention-dependent operations. **Objectives:** As *N*-methyl-d-aspartate receptor (NMDAR) hypofunction has been shown to contribute to these cognitive impairments, the primary aims of this healthy volunteer study were to: (a) to shed light on the separate and interactive roles of nAChR and NMDAR systems in the modulation of auditory sensory memory (and *sustained* attention), as indexed by the auditory event-related brain potential – mismatch negativity (MMN), and (b) to examine how these effects are moderated by a predisposition to auditory hallucinations/delusions (HD). **Methods:** In a randomized, double-blind, placebo-controlled design involving a low intravenous dose of ketamine (0.04 mg/kg) and a 4 mg dose of nicotine gum, MMN, and performance on a rapid visual information processing (RVIP) task of sustained attention were examined in 24 healthy controls psychometrically stratified as being lower (L-HD, *n* = 12) or higher (H-HD) for HD propensity. **Results:** Ketamine significantly slowed MMN, and reduced MMN in H-HD, with amplitude attenuation being blocked by the co-administration of nicotine. Nicotine significantly enhanced response speed [reaction time (RT)] and accuracy (increased % hits and *d*′ and reduced false alarms) on the RVIP, with *improved performance accuracy* being prevented when nicotine was administered with ketamine. Both % hits and *d*′, as well as RT were poorer in H-HD (vs. L-HD) and while hit rate and *d*′ was increased by nicotine in H-HD, RT was slowed by ketamine in L-HD. **Conclusions:** Nicotine alleviated ketamine-induced sensory memory impairment and improved attention, particularly in individuals prone to HD.

## Introduction

In patients with schizophrenia, the cardinal clinical (positive and negative) symptoms are, to variable degrees, accompanied by a wide range of neurocognitive impairments, particularly in the domains of attention and working memory. The various cognitive deficits are neither state-related nor specific to subtypes of the illness, but are considered as enduring features which remain relatively constant over the course of the disease and are at the very core of the dysfunction in schizophrenia patients (Eleväg and Goldberg, 2000; Heinrichs, 2005). As cognitive impairments in schizophrenia are highly correlated with functional (social and vocational) outcome, and both are relatively unaffected by dopamine-based antipsychotic drugs, pharmacotherapeutic initiatives are focusing on alternative molecular mechanisms as promising pharmacological targets for improving cognitive abilities in this disorder (Green, 2007; Tcheremissine et al., 2012).

Converging evidence from preclinical and human research points to the therapeutic potential of drugs targeted at nicotinic acetylcholine receptors (nAChRs) in the treatment of schizophrenia cognition (Ochoa and Lasalde-Dominicci, 2007; Evans and Drobes, 2009; D’Souza and Markou, 2011). nAChRs are strongly implicated in normal cognitive information processes (Mansvelder et al., 2006). Nicotine, the primary psychoactive chemical in tobacco smoke, is a prototypical nAChR agonist and the increased prevalence of smoking in schizophrenia (∼70–80%) compared to the general population (∼20%) has been interpreted as a form of self-medication (Kumari and Postman, 2005; Winterer, 2010), perhaps to compensate for reduced expression of nAChRs observed in post-mortem hippocampal and cortical brain regions of patients (Breese et al., 2000). *Although recent evidence of nicotine interacting with schizophrenia risk genes with regards to the expression of endophenotypes such as sensory gating may suggest that smoking might act as a causal factor for schizophrenia and related cognitive deficits* (Quednow et al., 2012), meta-analysis has shown significant enhancing effects of acute nicotine on multiple cognitive domains in healthy smokers and non-smokers (Heishman et al., 2010). Although null findings have been reported in the cognitive literature, acute doses of nicotine in animal models of schizophrenia and in non-smoking and smoking schizophrenia patients also transiently improve elementary pre-attentive sensory processing deficits as well as performance impairments in higher-order attention and working memory tasks (Olincy and Stevens, 2007; Radek et al., 2010).

Neurochemical models of schizophrenia have *implicated* glutamatergic mechanisms in general and *N*-methyl-d-aspartate receptors (NMDARs) in particular. NMDAR-type glutamate receptors are widely distributed throughout the brain and single sub-anesthetic doses of NMDAR antagonists, such as phencyclidine or ketamine, induce transient and reversible neurochemical, symptomatic and neurocognitive aspects of the disorder in healthy controls, with information processing deficits being observed not only in higher cortical regions, but also in subcortical systems and sensory cortices (Javitt, 2010; Adell et al., 2012). Over the past decade, bottom-up sensory processing deficits have become increasingly well documented in schizophrenia, co-existing with top-down, more complex forms of cognitive impairments and mirroring the pattern of deficits seen with NMDAR antagonists, thus supporting a NMDAR hypofunction model of the disease (Kantrowitz and Javitt, 2010).

Disturbance in low-level sensory problems in the auditory system is a robust cognitive deficit in schizophrenia patients and is strongly indexed by impaired generation of an event-related brain potential (ERP) component – the mismatch negativity (MMN). Generated within the primary auditory cortex and receiving contributions from frontal cortical generators (Rinne et al., 2000), the MMN is automatically elicited (at ∼150–200 ms) in an auditory “oddball” paradigm in response to infrequent changes (i.e., deviants, such as shifts in sound pitch, intensity, duration, location, or pattern) in a repetitive stream of auditory stimuli. Presumed to reflect the stored representations of the characteristics of auditory stimuli (for seconds to minutes), MMN is an index of auditory sensory (“echoic”) memory, a pre-attentive component of the brain working memory system (Naatanen et al., 2011). Impaired MMN generation, which is specific to schizophrenia (vs. other psychiatric disorders), is well established (Naatanen and Kahkonen, 2009), with a mean effect size of ∼1 d across studies (Umbricht and Krljes, 2005). Neither typical nor atypical antipsychotics affect the amplitude or latency of the ERP, and deficits in MMN generation have been reported in both clozapine- and risperidone-treated patients (Schall et al., 1998; Umbricht et al., 1998; Umbricht and Vollenweider, 1999; Kasai et al., 2002).

*N*-methyl-d-aspartate receptor antagonists have dose-dependently blocked the MMN response recorded in auditory primary cortex in animals (Javitt et al., 1994, 1996) and have diminished the MMN in healthy controls (Umbricht et al., 2000; Kreitschmann-Andermahr et al., 2001; Heekeren et al., 2008). Nicotine effects on MMN on the other hand have been relatively consistent with increased amplitude and shorter latencies to tone pitch deviants being observed in patients with Alzheimer’ disease (Engeland et al., 2002), whereas in healthy controls, nicotine has augmented MMN amplitudes to pitch (Harkrider and Hedrick, 2005), inter-stimulus interval (Martin et al., 2009), and duration deviants (Baldeweg et al., 2006), with the two former MMN effects being observed both in smokers and non-smokers, while the latter effect was shown only in smokers. The latency of the pitch-MMN has also been shortened with nicotine administration in non-smokers (Inauri et al., 2005), who have also evidenced a shortened latency and an increased amplitude of the pitch-MMN with an acute dose of the selective nAChR agonist AZD3480 (Dunbar et al., 2007), as well as an amplitude increase of the visual MMN with nicotine (Fisher et al., 2010).

In the first of two studies in schizophrenia patients, acute nicotine treatment did not alter the pitch-MMN amplitude in non-smoking patients and controls, but in the latter group, it shortened MMN latency relative both to placebo and to the MMN latency seen in the patient group (Inami et al., 2007). In the second study, conducted in our laboratory, nicotine also increased duration- (but not pitch) MMN in smoking patients, normalizing their amplitude relative to control smokers (Dulude et al., 2010). These effects were not associated with tobacco withdrawal symptoms or antipsychotic medication.

Nicotine modulates the release of neurotransmitters other than acetylcholine (e.g., *dopamine*, GABA, norepinephrine, and *glutamate*), with presynaptic nAChRs facilitating NMDAR-mediated glutamatergic neurotransmission in a multitude of brain regions including (but not limited to) the prefrontal cortex (Lambe et al., 2003). *nAChRs are heavily expressed in the primary auditory cortex* (Soto et al., 2006) *where nicotine has enhanced tone-evoked physiological sensitivity through NMDAR activation* (Metherate, 2004). Previous preclinical cognitive studies of nAChR-NMDAR interactions have shown mixed findings (Timofeeva and Levin, 2011), with some reporting nicotine blockade of the disruptive pre-attentive (sensorimotor gating), attentional, and mnemonic effects of NMDAR antagonists (Thompson and Winsaver, 1986; Terry et al., 2002; Rezvani and Levin, 2003; Spieleway and Markov, 2004; Andreasen et al., 2006; Levin and Rezvani, 2006; Rezvani et al., 2008), while others reported no interactions, or observed a potentiation of impairments induced with NMDAR antagonist treatment (Levin et al., 2003, 2005; Rezvani and Levin, 2003; Quarta et al., 2007; Rasmussen et al., 2008). In the few human studies, the NMDAR antagonist memantine did not oppose the smoking-induced improvements in sustained attention (Jackson et al., 2009), and in our work with ketamine, nicotine moderation of the arousal and attentional modulating actions of this NMDAR antagonist were found to be dependent on smoker vs. non-smoker status (Knott et al., 2006, 2011).

Mismatch negativity deficits in schizophrenia are highly correlated with cognitive and functional outcome and as the NMDAR antagonist model has shown predictive ability for a range of novel treatments that have reached clinical trials (Large, 2007), the human ketamine model, together with the use of putative endophenotypes such as MMN, offer a good opportunity to study new drugs with novel and distinct cognitive enhancing mechanisms that go beyond dopamine transmission. In the present study, the first to investigate the role of NMDAR-mediated glutamatergic neurotransmission in nicotine-modulated sensory memory, the separate and combined actions of nicotine and ketamine were examined with respect to auditory MMN. In addition, as NMDAR antagonism also impairs sustained attentional performance, typically slowing response speed and reducing accuracy in continuous performance tasks (CPT), a performance pattern similar to that observed in schizophrenia patients (Newcomer et al., 1999; Krystal et al., 2005a,b), the study also examined the effects of these drugs and their interactions on the Rapid Visual Information Processing (RVIP) task, a CPT with putative endophenotypic sensitivity (Hilti et al., 2010). As it is unclear as to whether ketamine-induced cognitive impairments reflect the direct effects of ketamine or are secondary to the induced schizophrenia-like clinical symptoms, we followed our previous studies and administered a sub-perceptual, non-psychotomimetic dose of ketamine (Knott et al., 2006, 2011).

Although the human ketamine model in healthy volunteers is well established and allows for the investigation of neurotransmitter systems participating in NMDAR-mediated cognitive deficiency, these findings may not necessarily be relevant to cognitive impairment in schizophrenia. Confounding factors (prior or concomitant drug treatment, chronicity, lack of cooperation, lower education) make the *in vivo* study of ketamine-drug interactions in schizophrenia difficult. However, the use of healthy surrogate populations (e.g., unaffected relatives of patients or people with schizotypal personality features), defined as groups that feature a component of the main disease process but do not have the fully developed condition – is gaining momentum as a potential methodology for detecting novel drug treatment for schizophrenia and as such may be relevant for capturing NMDAR-nicotinic interactions regulating cognitive endophenotypes of schizophrenia. In this approach, schizophrenia is viewed as an extreme of normally distributed cognitive functions and, for people who express some of the phenotype without the full disease (psychoses), their endophenotypes (such as MMN and RVIP) are thought to be more sensitive to drug effects and/or neurochemical disturbances than unselected healthy volunteers because they share the same elements of the disorder (Koychev et al., 2011). One phenotype, auditory hallucinations (AHs), is strongly associated with psychotic disorders such as schizophrenia but is also observed in other clinical and non-clinical groups (i.e., are “trans-diagnostic”). Increasingly, AHs are being investigated in non-psychotic conditions with cognitive and behavioral paradigms developed for schizophrenia (Waters et al., 2012). As the trait that makes humans prone to AHs appears to be related to brain areas involved in auditory stimuli processing/speech perception (i.e., auditory cortex; Kuhn and Gallinat, 2010), the same brain regions participating in MMN generation, and as schizophrenia patients with AHs (vs. those without AHs) have exhibited reduced MMN-indexed auditory sensory memory in our laboratory studies (Fisher et al., 2008, 2011, 2012a,b), this present investigation examined the effects of ketamine, nicotine, and their interactions in healthy volunteers psychometrically assessed as varying in hallucination/delusion (HD) proneness.

It was expected that individuals with higher (vs. lower) HD proneness would exhibit reduced MMN and RVIP performance, be more susceptible to the detrimental effects of ketamine, and be more responsive to the enhancing actions of nicotine when administered alone and in combination with ketamine.

## Materials and Methods

### Study participants

A sample of 24 participants (10 males, 14 females) was selected from a larger group of 38 healthy volunteers recruited from advertisements in local media and universities. All potential participants were screened via a semi-structured interview for psychiatric disorders (including alcohol/drug abuse) and general health, and were also assessed with the Family Instrument for Genetic Studies (Nurnberger et al., 1994) to rule out those with psychiatric disorders within first-degree relatives. They were also administered the Bell Object Relations and Reality Testing Inventory (BORRTI; Bell, 1992), a 90-item, self-report true-false pencil-and-paper questionnaire: 45 items assessing object relations, and the other 45 reality testing, with the latter yielding three subscale factors (Reality of Distortions, Uncertainty of Perceptions, and Hallucinations and Delusions), which are thought to identify those with a predisposition for psychotic symptoms. Only the HD subscale was used in this study. HD scores correlate significantly with the Hallucinatory Behavior and Unused Thought Content scales of the widely used Brief Psychiatric Rating Scale (BPRS; Overall and Gorham, 1962) and HD is the only subscale of the BORRTI on which high scores are specific to schizophrenia vs. other criterion groups (Bell et al., 1985). Standardized HD *t* scores of the total sample ranged from 30 to 67 (mean = 46.5) and those individuals with the 12 lowest (L-HD) scores (mean = 36.9, range 30–42) and the 12 highest (H-HD) scores (mean = 56.3, range 52–67) were selected for this study. A *t* score of 60 or more is indicative of a deficit and three of the H-HD group exhibited scores of 61, 61, and 67. L-HD and H-HD groups did not differ with respect to their scores on the Reality of Distortions and Uncertainty of Perception subscale factors. Groups were similar with respect to age, gender, and non-smoker/smoker status (L-HD: seven non-smokers, five smokers; H-HD: six non-smokers, six smokers). All the non-smokers had not smoked a cigarette in the past year, and the smoking characteristics of the smoking in the two groups was similar, with smokers smoking on average for 7.8 years (range 4–12) and smoking a mean of 16.8 cigarettes/day (range 15–20). None of these participants reported a personal or first-degree family psychiatric history, use of medications or a serious medical problem, and none exhibited abnormal results during a physical examination (with electrocardiogram) or with routine laboratory tests (complete blood count, blood chemistry, urine analysis, and urine toxicology for drug use).

The study was approved by the Research Ethics Board of the Royal Ottawa Health Care Group and all participants signed an informed consent prior to study participation.

### Experimental design

The study involved four test sessions couched within a double-blind, placebo-controlled, cross-over design with two parallel groups, L-HD and H-HD. Each session involved the intravenous infusion of ketamine (KI) or a comparable placebo (PI) as well as oral administration (gum) of nicotine (NG) or a comparable placebo (PG). Two of the four test sessions involved a KI and in the remaining two sessions participants received a PI. Also, in one of the KI and PI sessions, participants were pretreated with NG, and in the other two (KI and PI) sessions they were pretreated with PG. Order of the four treatments (PG–PI, PG–KI, NG–PI, NG–KI) was counterbalanced and sessions were separated by a minimum one-day interval.

### Session procedures

In each of the four test sessions, participants arrived at the laboratory (08:00 a.m.) following overnight (beginning 12:00 a.m.) abstinence from food, drugs, alcohol, caffeine, and cigarettes. Smoking abstinence was verified by an expired air carbon monoxide (CO) reading, which was required to be below 5 parts per million (ppm). Following insertion of an antecubital intravenous line, a 45-min adaptation period and EEG electrode placement, participants were administered either NG or PG, and after a 30-min nicotine absorption period, KI or PI was initiated along with the RVIP task and simultaneous ERP recordings for MMN. Vital signs were assessed both before drug treatment and at the end of the session.

### Drug administration

#### Nicotine

An oral dose of nicotine was administered in the form of nicotine polacrilex gum, providing delivery primarily *via* buccal absorption (Hukkanen et al., 2005). A 4 mg Nicorette Plus (Hoechst Roussell) gum piece or matching placebo was chewed (with nose plugs, to help reduce any possible sensory impact differences between nicotine and placebo) over a 25-min period according to manufacturer guidelines, with participants using an audiotape to instruct them to bite the gum twice per minute and to *park* the gum (i.e., inside the mouth between teeth and cheeks) after each bite. An additional 5-min absorption period, involving the chewing of a strong mint gum to help disguise any placebo vs. nicotine flavor differences, followed the gum chewing so that peak blood levels from the subsequent infusion of ketamine coincided with slower rising blood nicotine concentrations, which typically exhibit a *T*_max_ of ∼30-min and are expected to range between 10 and 17 ng/ml (Hukkanen et al., 2005). Plasma levels of nicotine were not assessed. The elimination half-life of plasma nicotine is approximately 120 min.

#### Ketamine

Human ketamine studies of acute psychoses typically involve systemic infusions of ketamine at dose levels well below the 1–2 mg/kg dose range used in human anesthesia, either by administering a constant dose (0.5 mg/kg) over a ∼60-min period (Krystal et al., 1994), or by administering an initial bolus (Malhotra et al., 1996) or loading dose (Newcomer et al., 1999), both followed by infusion of a maintenance dose of ketamine to achieve steady state ketamine blood levels over a ∼60-min period. As loading doses of 0.27 and 0.08 mg/kg have produced marked and mild psychotomimetic-like reactions, respectively, with no subjective effects or measurable plasma ketamine levels being observed with 0.024 mg/kg (Newcomer et al., 1999), this study utilized an intermediate low loading dose of 0.04 mg/kg, but no maintenance dose, with the aim of avoiding the experience of schizophrenia-like clinical symptoms. *Although there are significant relations between MMN Amplitudes and psychoses-like symptoms induced by ketamine* (Umbricht et al., 2002), *our use of a relatively low dose allows us to study the effects of ketamine independent of psychotic symptoms*. An automated pump apparatus (Imed Gemini PC-1) infused 0.04 mg/kg ketamine hydrochloride or placebo [saline (0.9% sodium chloride) solution] over a 10-min period in order to maximize safety. This dose level, previously used in our laboratory, had been associated with arousal EEG, and cognitive changes in healthy volunteers (Knott et al., 2006, 2011) and produced ketamine blood levels (25–37 mg/ml) that equated with levels seen in a prior study that administered a 0.08 mg/kg dose of ketamine (Newcomer et al., 1999). Although not able to be assessed in all the study participants due to prohibitive costs, gas chromatography (National Medical Services, Philadelphia, PA, USA) carried out on five plasma samples obtained from the PG–KI condition (collected at termination of the infusion) found KI to produce ketamine levels ranging from 25 to 37 ng/ml (*M* = 31 ng/ml). These ketamine levels approximate the average ketamine level reported with the 0.08 mg/kg dose of ketamine (Newcomer et al., 1999).

### Study assessments

In order to minimize attention to the auditory stimuli eliciting the MMN, these ERPs are typically acquired while participants are engaged in a secondary visual task, which in this case, was the RVIP task.

#### RVIP paradigm

Employing the original RVIP paradigm of Wesnes and Warburton (1984), participants viewed 50-ms duration single digits (1–9) presented (black on white) in the center of a monitor at a fixed rate of 110 digits/min, the average inter-stimulus interval being 545 ms and ranging between 445 and 645 ms. Button presses were required on the detection of targets, which were defined as the third of three consecutive odd digits. Over the 12-min task, a total of *1320* digits, *including 120 targets* (10 per min) were presented, with each target sequence being separated by a minimum of five digits. Volunteers were instructed to respond (with right index finger) as quickly and accurately as possible and performance measures included: the number of correct responses (expressed as % hits), with correct responses being defined as a button press to the third digit of each target triad within 100–1000 ms post-stimulus onset; the number (%) of incorrect or false alarm (FA) responses, defined as button presses to non-target stimuli, and reaction times (RT) of correct responses. *In addition, signal detection methodology was used to equally weight hits and FAs into a single measure (d′), which is considered to be a purer index of perceptual accuracy/sensitivity* (Macmillan and Creelman, 1997). Response measures were separated into three successive time blocks of 4-min each to assess performance changes with drugs across time.

#### MMN paradigm

Auditory stimuli eliciting the MMN during ERP recordings consisted of a total of 1200, 80 dB tones (50 ms duration 10 ms rise/decay time) presented binaurally through headphones (80 dB sound pressure level) with a stimulus onset asynchrony of 545 ms (ranging between 445 and 645 ms) and between individual RVIP stimuli; resulting in no temporal overlap of the two stimulus modalities. Ten percent of the tones (deviants) were 100 Hz and were randomly presented among the standard tones (1000 Hz). Electrical activity was recorded from frontal (F*_z_*), central (C*_z_*), and parietal (P*_z_*) midline scalp sites *using a* linked earlobe *reference*. *Although choice of reference site may potentially alter outcomes, ketamine-induced reductions in MMN have been observed with a variety of references, including linked ears* (Gunduz-Bruce et al., 2012) *and linked mastoids* (Umbricht et al., 2000). *Recordings were also taken* from orbital sites *and* amplifier bandpass filters and EEG sampling rate set at 0.1–40 Hz and 250 Hz, respectively. ERP processing included epoch segmentation (500 ms, beginning 50 ms pre-stimulus onset), ocular correction, artifacting (eliminating ocular corrected epochs with EEG > 100 μV), baseline correction, and separate averaging of standard and deviant epochs. As with the RVIP data, averages were separated into three equal time blocks, with each block containing 400 stimuli (40 deviants). There were no significant differences between the study conditions in terms of the number of epochs comprising standard and deviant averages and all deviant averages contained a minimum of 30 stimuli. MMNs were derived by digital point-by-point subtraction of standard waveforms from deviant waveforms. *Since out own work found frontal MMN in schizophrenia to be effected by acute nicotine* (Dulude et al., 2010) *and as ketamine-induced reductions in MMN in healthy volunteers was evidenced only at frontal (vs. temporal) sites* (Schmidt et al., 2012a), *the* MMN was measured from F*_z_*, the site of maximal amplitude. Based on grand averages, *MMN amplitude* was defined as the peak negative voltage (relative to pre-stimulus baseline) in an 80–220 ms window. Latency (ms) was measured as the time (from stimulus onset) to reach peak negativity.

### Statistical analysis

Performance measures (hits, FA, *d*′, RT) were subjected to separate split-plot ANOVAs with one between-group factor (HD, two levels) and three within-group factors (gum, two levels; drug, two levels; time block, three levels). Similar ANOVAs were carried out with the ERP measures (MMN amplitude and latency). Greenhouse–Geisser corrections were applied where appropriate to compensate for violations of sphericity assumed with univariate ANOVAs. Significant (*p* < 0.05) main or interaction effects were followed up with Bonferroni adjusted pairwise comparisons.

*Exploration of relationships between MMN and sustained attention were examined with Pearson r correlations between MMN amplitude and d′ (collapsed across groups and time blocks) using placebo data (i.e., PG plus PI condition) as well as change score data, derived by subtracting values in the placebo condition (PG–PI) from values derived in each of the three non-placebo sessions*.

## Results

### Effects on MMN

**Figure 1:**
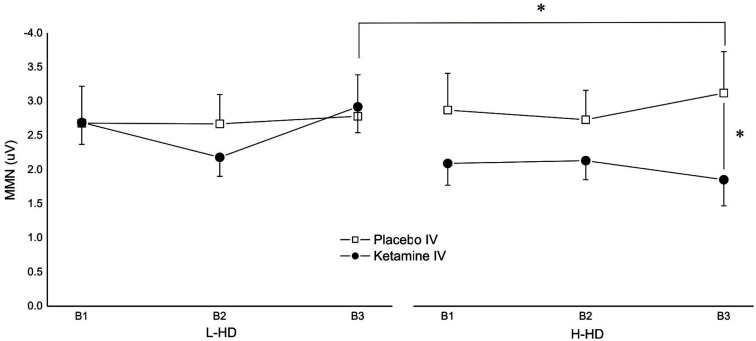
Effects of placebo and ketamine infusion (collapsed across placebo and nicotine gum) on frontal (F*z*) mean (± SE) MMN amplitudes of L-HD and H-HD participants in the three time blocks (B1–B3; *p < 0.05).

Robust MMN amplitudes to pitch deviants were shown in each of the four test sessions and in each of the three time blocks within each session. No significant main effects were observed for drug, gum, group, or time block but a significant group × drug × block (*F* = 4.05, d*f* = 2, 44, *p* = 0.031) interaction was demonstrated for amplitudes. Although exhibiting a general amplitude reduction with KI, planned comparisons showed that compared to placebo, KI (vs. PI) significantly reduced MMN only during time block 3 in the H-HD group (*p* = 0.038). In addition, the MMN amplitude of the H-HD group during time block 3 was significantly smaller (*p* = 0.05) than that of the L-HD group during KI (Figure 1).

**Figure 2:**
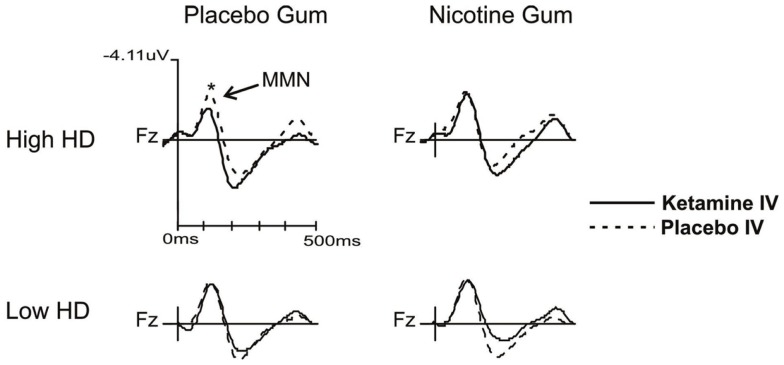
Grand averaged waveforms showing MMN changes (collapsed across time blocks and L-HD and H-HD groups) with ketamine and placebo infusions when combined with nicotine and placebo gum (*p < 0.05).

Follow-up analysis of a significant group × drug × gum interaction (*F* = 3.945, d*f* = 1, 22, *p* = 0.049) showed a significant ketamine effect in the H-HD group. More specifically, when administered with PG, KI (vs. PI) significantly (*p* = 0.004) attenuated MMN amplitudes of the H-HD participants (Figure 2). Additionally, in this same condition when KI was combined with PG, the H-HD exhibited significantly smaller MMN amplitudes than the L-HD group (*p* = 0.004). In the H-HD group, KI did not attenuate MMN when it was administered with NG (i.e., nicotine blocked KI effects on MMN) and MMN was shown to be significantly smaller when KI was combined with PG compared to when it was combined with NG (*p* = 0.007).

**Figure 3:**
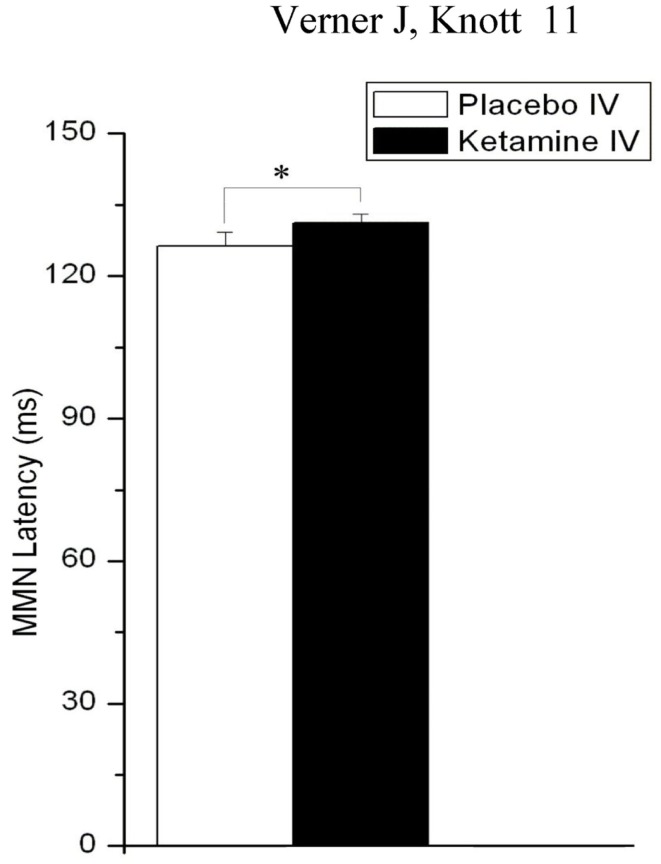
Effects of placebo and ketamine infusion (collapsed across gum conditions, time blocks, and L-HD and H-HD groups) on mean (± SE) frontal (F*z*) MMN latency (*p < 0.05).

In addition to amplitude, latency of MMN was slightly but significantly affected by drug (*F* = 4.38, d*f* = 1, 22, *p* = 0.048), with KI resulting in a slowing of MMN relative to PI (Figure 3).

### Effects on RVIP

**Figure 4:**
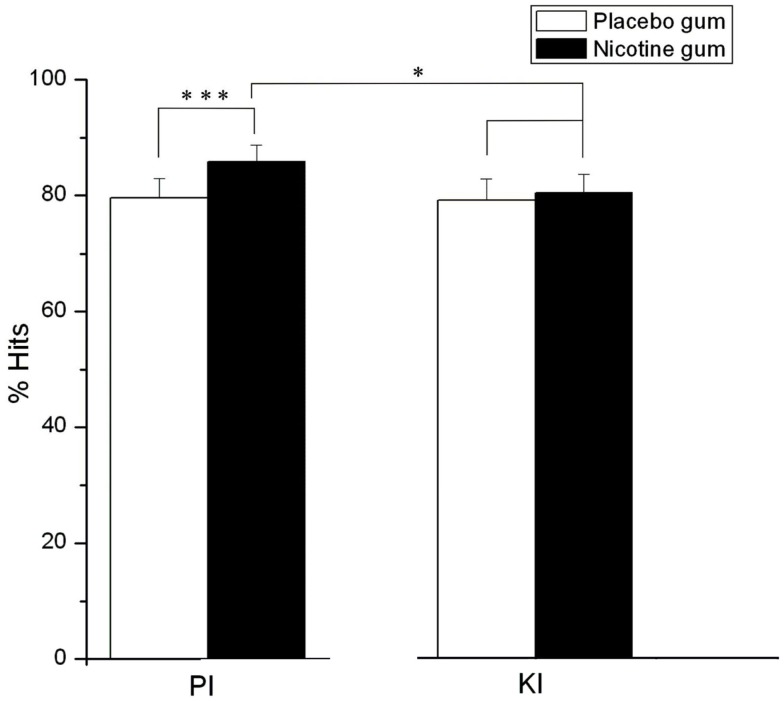
Effects of placebo and nicotine gum (collapsed across time blocks, and L-HD and H-HD groups) on mean (± SE)% hits during placebo (PI) and ketamine (KI) *infusion* (*p < 0.05; ***p < 0.001).

Response accuracy as expressed by % hits varied significantly in relation to time block (*F* = 5.27, d*f* = 2, 44, *p* = 0.01), with hits being reduced (*p* = 0.01) in time blocks 2 (*M* = 79.94%, SE ± 4.98) and 3 (*M* = 79.16%, SE ± 5.18) compared to time block 1 (*M* = 84.38%, SE ± 3.76). A significant gum (*F* = 11.15, d*f* = 1, 22, *p* = 0.003) and a gum × drug interaction (*F* = 10.05, d*f* = 1, 22, *p* = 0.004) demonstrated a general enhancing effect of NG (vs. PG) on target detection, which was blocked by KI. Specifically, in the PI conditions, NG significantly elevated % hits relative to PG (*p* = 0.0001) but this gum effect was not observed under KI conditions (Figure 4). The blockade of nicotine-induced increases in hit rates was further reflected in the comparison of the two NG conditions, where hits during the combined NG-KI condition were significantly reduced compared to when nicotine was combined with PI (*p* = 0.05) *and when KI was administered alone (p* < 0.05).

**Figure 5:**
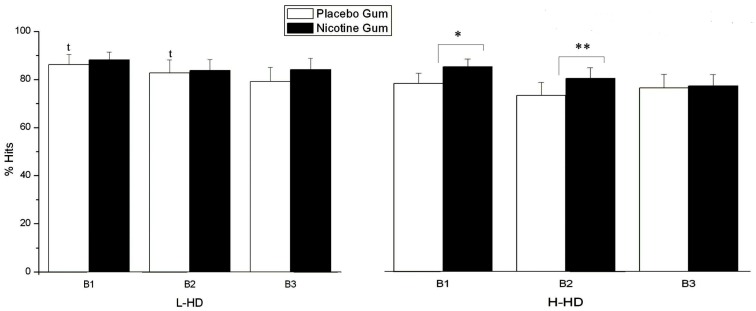
**Effects of placebo and nicotine gum (collapsed across placebo and ketamine infusions) on mean (±SE) % hits of L-HD and H-HD groups in the three times blocks (B1–B3)**. (*p < 0.006; **p < 0.001; *t*, *p* < 0.05 comparing L-HD and H-HD with placebo gum in B1 and B2).

In a group × gum × block interaction (*F* = 3.64, d*f* = 1, 44, *p* = 0.046), the H-HD group exhibited significantly (*p* = 0.04) reduced % hits than the L-HD group in time block 1 (*p* = 0.05) and time block 2 (Figure 5). For the H-HD group, NG (relative to PG) was shown to elevate hit rate in the H-HD during time block 1 (*p* = 0.006) and during time block 2 (*p* = 0.001).

**Figure 6:**
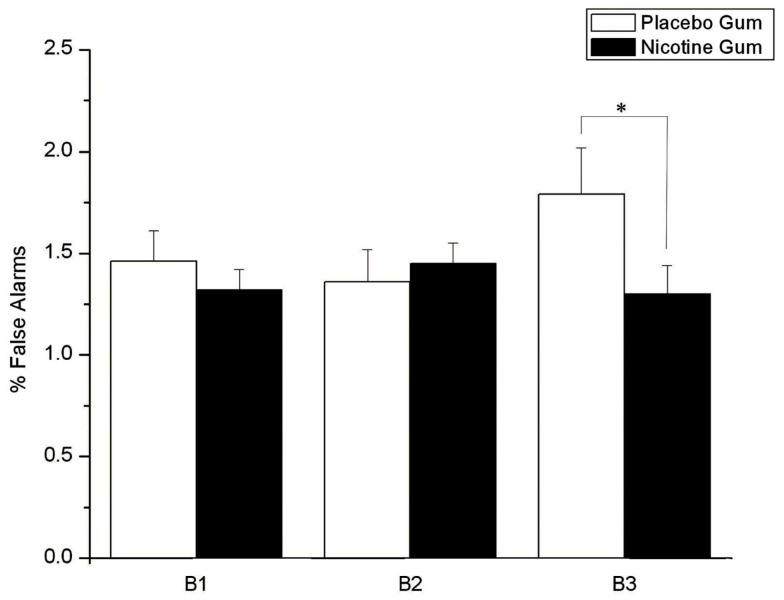
Effects of placebo and nicotine gum (collapsed across placebo and ketamine infusions, and L-HD and H-HD groups) on mean (±SE) % false alarms in the three time blocks (B1–B3; *p < 0.05).

As shown in Figure 6, FAs were moderated by a gum × time block interaction (*F* = 3.23, d*f* = 2, 44, *p* = 0.032), with NG acting to prevent the response errors seen in time block with PG (*p* = 0.038).

The analysis of *d*′ yielded a significant gum (*F* = 13.63, d*f* = 1, 44, (*p* = 0.001), drug × gum (*F* = 7.06, d*f* = 1, 44, (*p* = 0.014) and a drug × gum × block × group effects (*F* = 3.64, d*f* = 1, 44, (*p* = 0.05). NG (vs. PG) increased scores (*p* = 0.001) but this was shown only during PI (not KI) and, as displayed in Table 1, was limited to the H-HD group during time block 1 (*p* = 0.001) and 2 (*p* = 0.009). During administration of placebo (PG–PI), *d*′ scores of the H-HD group were also significantly lower than those of the L-HD group in time block 1 (*p* = 0.05) and 3 (*p* = 0.05). Also for the H-HD group in the combined Ki-NG condition, *d*′ scores significantly declined from block 1 to block 2 (*p* = 0.01).

**Table 1:** **Mean/±SE *d*′ scores of L-HD and H-HD groups across treatment conditions and time blocks (B1–B3)**.

Treatment	L-HD			H-HD		
	B1	B2	B3	B1	B2	B3
PG–PI	3.49/0.18	3.45/0.27	3.39/0.28	3.10/0.18	3.02/0.27	2.95/0.28
PG–KI	3.44/0.23	3.38/0.24	3.04/0.29	3.26/0.23	3.04/0.24	3.38/0.29
NG–PI	3.66/0.19	3.62/0.24	3.49/0.25	3.86/0.19	3.27/0.24	3.44/0.25
NG–KI	3.46/0.21	3.23/0.2	3.44/0.25	3.26/0.21	3.29/0.22	3.05/0.25

**Figure 7:**
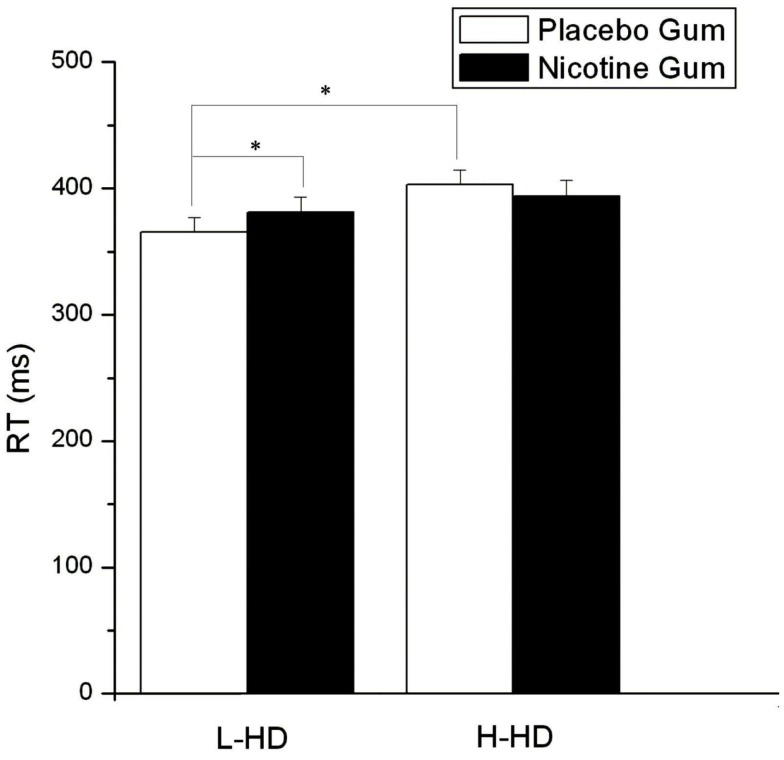
Effects of placebo and nicotine gum (collapsed across placebo and ketamine infusions and the three time blocks) on mean (±SE) reaction time (RT) in L-HD and H-HD groups (*p < 0.05).

Response speed was significantly influenced by gum (*F* = 4.77, d*f* = 1, 22, *p* = 0.040), with NG speeding RT relative to PG. In a group × drug interaction (*F* = 4.77, d*f* = 1.22, *p* = 0.040) RT of the H-HD group was found to be significantly slower (*p* = 0.032) than the L-HD during administration of PG and PI (Figure 7). Although not affecting the H-HD participants, *there was a trend for* KI (vs. PI) *to slow* RT (*p* = 0.06) in the L-HD group, and during the KI condition there were no differences in RT between the L-HD and H-HD groups.

### Correlations

*No significant correlations were observed between MMN and d′ in the placebo condition (PG–PI) nor were there any significant correlations between ketamine/nicotine-induced changes in MMN and ketamine/nicotine-induced change in d′*.

## Discussion

To our knowledge, this is the first study to investigate the moderating role of nicotine on ketamine-induced changes in both sensory and attentional processing, two domains which are highly dysfunctional in schizophrenia and are differentially modulated by the NMDAR antagonist ketamine and the nAChR agonist nicotine. Specifically, the study investigated the hypothesis that MMN – an index of auditory sensory memory that depends on intact NMDAR functioning, would be impaired by ketamine but not when co-administered with nicotine. The results support this hypothesis in that healthy volunteers, assessed to be more prone to experiencing hallucinatory activity (H-HD), evidenced a significantly diminished MMN generation with ketamine alone but not when ketamine was combined with nicotine. During ketamine infusion, MMN amplitudes of H-HD participants were significantly reduced compared to L-HD participants, assessed to be less prone to HD.

Although not observed in one previous study (Oranje et al., 2000), the reduction of MMN with intravenous ketamine is consistent with prior reports of MMN attenuation in healthy volunteers with sub-anesthetic doses of the NMDA antagonist ketamine (Umbricht et al., 2000) and parallels the reduction of MMN in monkeys following infusion of NMDAR antagonists into primary auditory cortex (Javitt et al., 1992, 1994, 1996) as well as the dose-dependent blockade of the MMN recorded from rat auditory cortex after intra-peritoneal injections of the NMDAR antagonist, MK-P01, and memantine (Tikhonravov et al., 2008, 2010). Together with the observation of robust reductions of MMN (effect size of 0.99) in schizophrenia patients (Umbricht and Krljes, 2005) and the wealth of evidence suggesting that psychosis is secondary to NMDAR hypofunction with a downstream effect in dopaminergic activity (Fu et al., 2000; Lin et al., 2012), these present findings provide indirect evidence supporting the glutamate/NMDAR hypothesis of schizophrenia and specifically reinforce the implication that deficits in glutamatergic signaling underlie auditory sensory memory impairment characterizing this disorder.

Unlike previous cognitive and MMN studies that purposefully used ketamine doses which were psychotomimetic (i.e., typically involving initial loading dose of ∼0.25 mg/kg followed by a maintenance dose of ∼0.5–0.9 mg/kg/h) and transiently reproduced schizophrenia-like negative and positive symptoms in healthy volunteers (e.g., Newcomer et al., 1999), this present study used a single, low dose bolus infusion (0.04 mg/kg) which had previously been shown to induce only mild subjective arousal and euphoric but not clinically relevant behavioral changes. MMN is indirectly proportional to the prevalence of negative symptoms (Urban et al., 2007) and as such, ketamine-induced behavioral effects with psychotomimetic doses may potentially contribute to cognitive deficits including sensory processing abnormality. However, in healthy controls administered the psychotomimetic psilocybin (a 5-HT2A agonist), a reduction in MMN was not shown despite the experienced marked behavioral changes (Umbricht and Vollenweider, 1999). Viewed in the context of previous reports of greater MMN deficits in patients prone to AH (Fisher et al., 2008, 2011, 2012a,b) and of studies showing the magnitude of MMN deficit in patients to be correlated with clinical ratings of hallucinatory behavior (Youn et al., 2003; see also, Hirayasu et al., 1998 and Thonnesen et al., 2008), our finding of diminished MMN generation with a low sub-psychotomimetic dose of ketamine in H-HD (vs. L-HD) participants suggests that glutamatergic neurotransmission in the auditory cortex of individuals with a trait that predisposes to AH may be more vulnerable to disruption of NDMAR activity.

Keeping in mind that AHs of non-patients may not be similar to those of psychotic or non-psychotic clinical patients (Daalman et al., 2011), hallucinations in general are essentially perceptions that arise through an interaction stemming from neural activations and top-down activity. The basic neural signal contributing to a tonic trait-like vulnerability to experience AHs appears to arise from hyperactivation of functional networks involving the auditory cortex that generate aberrant auditory signals (Kuhn and Gallinat, 2010; Waters et al., 2012). Although AHs were not formally assessed during the testing sessions, it is possible that NMDAR antagonism induced an abnormality in auditory signaling and increased hallucinating activity, which may have impacted MMN generation. In support of this argument, patients in general exhibit reduced sensory-level processing of auditory input (e.g., diminished auditory acuity, elevated thresholds for tone discrimination; Mathew et al., 1993; Holcomb et al., 1995; Strous et al., 1995; Wexler et al., 1998; Rabinowicz et al., 2000); patients with (vs. without) AHs have greater difficulty in sound (speech) discrimination (Hugdahl et al., 2008), and the temporal cortex evidences both high glutamate levels in patients (Marsman et al., 2011) and hypofunctional activation in controls during ketamine infusion (Hugdahl et al., 2008). However, impaired auditory MMN generation during ketamine infusion is unlikely to be related to competing influences resulting from increasing auditory verbal hallucinations as organized AH (i.e., identifiable as speech) as seen in established schizophrenia are rarely observed during a ketamine challenge (Kantrowitz and Javitt, 2010). Also, any evoked auditory disturbances during ketamine infusion resemble the pattern observed in the early course of schizophrenia and as such, altered auditory processing during a ketamine challenge may be viewed more as a model of prodromal or acute incipient schizophrenia, rather than late, chronic schizophrenia (Kantrowitz and Javitt, 2010).

Nicotine alone did not affect MMN but it prevented the MMN attenuation induced with ketamine. Failure to observe enhanced sensory memory processing with nicotine as shown in earlier reports may be related to methodological differences between studies, including dose and route of nicotine administration, stimulus feature and presentation parameters, ERP recording and processing procedures, and composition of participant samples. However, the ability of nicotine to block the NMDAR antagonist actions of ketamine on auditory sensory memory is consistent with findings that acetylcholine is one of the major modulators of auditory cortical activity (Edeline, 2003; Soto et al., 2006), with both high (α4β2) and low (α7) nicotine affinity nAChRs being densely, and widely expressed in the auditory pathway (Morley and Happe, 2000), particularly where thalamocortical inputs terminate (Metherate, 2004; Bieszczad et al., 2012).

The underlying mechanism of nicotine’s protective action is unclear and although it may involve the regulation of sensory processes by tonic release of endogenous acetylcholine at nAChRs, thalamocortical synapses are excitatory and glutamatergic (Kharazia and Weinberg, 1994). Hence, nicotine blockade of ketamine effects may implicate nicotinic regulation of thalamocortical glutamate release by presynaptic (or preterminal) nAChRs, and possibly nicotinic regulation of GABAergic interneurons (Radcliffe et al., 1999; Schilström et al., 2000; Metherate and Hsieh, 2003; Liang et al., 2008; Intskirveli and Metherate, 2012). As an additional or alternative mechanism, nicotinic agonists have also been shown to inhibit NMDAR-mediated cortical currents, possibly by displacing the obligatory NMDAR co-agonist glycine (Flores-Hernandez et al., 2009). Regardless of the mechanism of action, these present study findings of diminished ketamine-induced MMN impairment were observed with a smoking-dose of nicotine, as was the previous finding of nicotine-induced normalization of MMN in schizophrenia patients (Dulude et al., 2010). Together they provide tentative support for the contention that excessive tobacco use in schizophrenia in patients may be an attempt to correct sensory deficiencies related to dysfunctional nAChR and/or NMDAR systems. The increase in MMN latency with NMDAR antagonism, reported previously by other studies using larger ketamine doses (Umbricht et al., 2000; Kreitschmann-Andermahr et al., 2001; Roser et al., 2011), was not moderated by nicotine, suggesting that nicotine’s protective actions in the human ketamine model are specific to the strength and not the speed of sensory memory processes.

Performance in the RVIP task was also investigated and was generally found to be enhanced during acute nicotine administration, with both response accuracy *(hit rate and d′)* and speed (RT) being improved relative to placebo. Indexing sustained attention and working memory aspects of cognition, task performance in CPT paradigms such as RVIP has consistently been impaired both in schizophrenia (Hilti et al., 2010; Barch et al., 2012) and tobacco abstaining smokers (Heishman, 1999). Nicotine has been shown to improve CPT efficiency in schizophrenia (Smith et al., 2006; Barr et al., 2008), with attentional improvements in some studies being reported to be selective for non-smoking (vs. smoking) patients (Harris et al., 2004). *For sustained attention measured with RVIP, nicotine improved but did not normalize performance in schizophrenia and it exerted no significant reversal of the impaired frontal-parietal-cingulate-thalamic attention network associated with schizophrenia* (Hong et al., 2011). Although typically viewed as a task of sustained attention, RVIP also involves a significant working memory component and nicotine improvement of RVIP and attention-dependent tasks may be related to its demonstrated ability to activate posterior (parietal) brain areas traditionally associated with visual attention (Lawrence et al., 2002) and/or to increase functional connectivity in frontal, executive-based cortical regions (Jacobsen et al., 2004).

The increased hit rate and *d*′ seen with nicotine (vs. placebo) gum during placebo infusion was absent when nicotine was administered with ketamine. Ionotropic- but not metabotropic-glutamate antagonist treatment also blocked nicotine improved response accuracy in rodent attentional tasks (Quarta et al., 2007; Amitai and Markou, 2009). Together these observations suggest that nAChR modulation of glutamate release underlies nicotine-enhanced target detection and is diminished by NMDAR blockade. However, nAChR and NMDAR ligands appear to overlap in their ligand affinities (Aracava et al., 2005; Plazas et al., 2007) and as anesthetics are potent inhibitors of presynaptic nAChRs, with ketamine being shown to block α4 β2 nAChRs (Irnaten et al., 2002; Tassonyi et al., 2002), it may have attenuated the nicotinic stimulation required for improvements in correct response rates.

Nicotine-facilitated target detection was found to vary between HD groups, with increased *d*′ and hit rates during nicotine (vs. placebo) administration being limited to the higher scoring individuals (H-HD), who also exhibited reduced hit rates and *d*′ compared to L-HD participants in the non-drug condition. Although in schizophrenia hallucinators, the reduced activation shown in primary auditory cortex during auditory target detection was not seen in visual cortex (Ford et al., 2009), recent neurophysiological evidence has pointed to early visual processing deficits in patients who tend to hallucinate (Kayser et al., 2012). Perhaps extending to healthy controls with a hallucinating propensity, these findings in patient hallucinators suggested a broader early perceptual deficit that extends beyond the auditory modality. The performance improving effect of nicotine on RVIP in H-HD is consistent with previous ERP research showing preferential enhancement in visual (vs. auditory) processing (e.g., increased amplitude of the N100 and P300 ERPs) with smoking/nicotine (Knott, 1989; Knott et al., 1995, 1999; Pritchard et al., 2004) and it also parallels reports showing nicotine – induced cognitive facilitation to be more prevalent in poorer performing, cognitively deficient populations (Newhouse et al., 2004).

The H-HD group were also slower in responding to RVIP targets than the L-HD group but ketamine, not nicotine, influenced response speed only in the L-HD participants, increasing RT relative to placebo. Together with reduced target detection rates, response slowing in non-patient hallucinators support and extend the contention applied to schizophrenia voice-hearers (Ford et al., 2009), that their auditory cortex may be “turned on” and tonically “tuned in” to internal acoustic information at the cost of processing not only external sounds but on the basis of these findings, external visual input as well. Perhaps reflecting a “floor” effect that prevents further slowing of RT, ketamine-induced slowing was not seen in H-HD and its appearance in L-HD indicates that, as shown with nicotine’s *attentional* enhancing properties, the *RVIP* impairing actions of NMDAR blockade in healthy volunteers are baseline dependent. *MMN failed to show any relationship to d′ in the placebo or active conditions. In schizophrenia, MMN has been found to be related and contribute to higher-order cognitive impairments as well as deficits in social cognition* (Javitt et al., 1995; Baldeweg et al., 2004; Sehatpour et al., 2010; Wynn et al., 2010). *Unlike our present findings*, *modeling study suggested ketamine reductions in MMN in healthy volunteers to be mediated by changes in short-term plasticity (of “forward” inter-regional connections) of the auditory hierarchy, which significantly correlated with ratings of ketamine-induced impairments in cognition and control (Schmidt et al., 2012b). Although RVIP may be an exception, re-examination of this task in larger sample of healthy volunteers and patients and with larger doses of nicotine and ketamine may show unique relationships between MMN and sustained attention that are moderated by SZ pathology and/or nicotinic/glutamatergic activity*.

### Limitations

Methodological weakness in the present study may have moderated the findings and limited the conclusions and implications of this research. First, both nicotine and ketamine were administered as single doses and accordingly, it is not know if the observed results with each drug and their combination are dose-dependent. Second, ketamine was infused as a low bolus dose and the related cognitive findings may be distinct from those observed with the majority of human ketamine studies that have assessed cognition with sustained psychotogenic doses of this drug. Also, blood levels of the two drugs were not assayed to determine bioavailability and the cognitive effects of nicotine administered via slow buccal absorption may not mirror the effects of acute smoking, which delivers rapid nicotine boli to the brain. *Performance* assessments were also limited and did not encompass the range of information processing deficits that characterize schizophrenia, each of which are valid targets for novel nicotinic and glutamatergic therapies. Finally, the L-HD and H-HD study samples were relatively small *and* were drawn from a healthy population and although there are advantages to this approach, similar challenge studies are required with healthy surrogate populations (e.g., schizotypal personality disorder, unaffected first-degree relatives of patients) with genetic links to schizophrenia.

## Conclusion

This study produced novel findings which underscore the potential role of nAChR and NMDAR systems and their interplay in the etiology of core neurocognitive deficits characterizing schizophrenia. The disturbance in sensory and attentional processing evident with a sub-psychotomimetic dose of ketamine reflects the widespread distribution of brain NMDARs and their sensitivity to minimal perturbations in glutamate signaling. Nicotine exhibited strong *attentional enhancing* properties, which in part were dependent on glutamate neurotransmission and were also evident by its ability to counter *impaired deviance detection* induced with NMDAR blockade. That these effects were moderated by a hallucinatory trait in a healthy population suggests that nicotine’s sensory-*attentional* properties and its modulatory effects on NMDAR systems are relevant to neurocognitive deficits in schizophrenia and may be specific to patient subtypes.

## Conflict of Interest Statement

The authors declare that the research was conducted in the absence of any commercial or financial relationships that could be construed as a potential conflict of interest.
